# Biocompatible Germanium-Enriched Nanocomposite Coatings on Titanium Designed Toward Preventing Early Inflammation and Promoting Osteogenic Differentiation

**DOI:** 10.3390/jfb17090464

**Published:** 2026-09-10

**Authors:** Miloš Lazarević, Evelina Herendija, Milica Jakšić Karišik, Marijana R. Pantović Pavlović, Miroslav M. Pavlović, Katarina R. Pantović Spajić, Nenad L. Ignjatović

**Affiliations:** 1 School of Dental Medicine, University of Belgrade, Dr Subotica 8, 11000 Belgrade, Serbia; milica.jaksic@stomf.bg.ac.rs; 2 Biomedical Engineering and Technology, Multidisciplinary PhD Studies, University of Belgrade, Studentski Trg 1, 11000 Belgrade, Serbia; nherendija@yahoo.com; 3 Department of Electrochemistry, Institute of Chemistry, Technology and Metallurgy, University of Belgrade, Njegoševa 12, 11000 Belgrade, Serbia; m.pantovic@ihtm.bg.ac.rs (M.R.P.P.); miroslav.pavlovic@ihtm.bg.ac.rs (M.M.P.); 4 Institute for Technology of Nuclear and Other Mineral Raw Materials, University of Belgrade, Franchet d’Esperey Street 86, 11000 Belgrade, Serbia; k.pantovic@itnms.ac.rs; 5 Institute of Technical Sciences of the Serbian Academy of Science and Arts, Kneza Mihaila 35/IV, P.O. Box 377, 11000 Belgrade, Serbia

**Keywords:** germanium-enriched coatings, double-action potential, dental pulp stem cells, immunomodulation, osteogenic differentiation

## Abstract

The study seeks to develop a multifunctional germanium-enriched nanocomposite coating on titanium and to evaluate its ability to modulate early inflammatory responses while promoting osteogenic differentiation in a dental pulp stem cell (DPSC)-based regenerative model. A multifunctional Ti/Coating composed of nanohydroxyapatite (nHAp) particles, chitosan-oligolactate (ChOL), and germanium (Ge) was developed using a combined anodizing/anaphoretic electrodeposition approach. The Ti/Coating system exhibited good biocompatibility, as confirmed by microscopy, 3-(4,5-dimethylthiazol-2-yl)-2,5-diphenyltetrazolium bromide (MTT) and lactate dehydrogenase (LDH) assays, with cell viability consistently exceeding 90% and moderate LDH release across all time points. Annexin V/PI assay demonstrated a predominance of viable cells (>94%), while intracellular ROS analysis indicated moderate oxidative activity. Gene expression analysis revealed significant downregulation of pro-inflammatory markers (*TNF-α*, *IL-1β*, *IL-6*, *COX-2*, and *MAPK*), suggesting attenuation of inflammatory signalling. Flow cytometry further demonstrated reduced CD120b (TNFR2) expression, while intracellular TNF-α levels remained unchanged, indicating selective modulation at the receptor level. In addition, the Ti/Coating promoted osteogenic differentiation, as evidenced by enhanced mineralization, and upregulation of osteogenic genes (*ALP*, *RUNX2*, *BMP2*).

## 1. Introduction

Inflammation plays a crucial role in the early biological response to titanium (Ti) implant treatment, directly influencing immune response, osseointegration and long-term clinical success. Immediately after implantation, host tissues activate innate and adaptive immune responses that consequently regulate the cascade of events at the bone–implant interface and determine the final healing outcome [1]. Therefore, recent biomaterials research has focused on immunomodulatory surface strategies aimed at optimizing an early inflammation microenvironment promoting tissue repair and stimulating the pro-regenerative action of the immune system [2]. In this context, the purpose of a nano-based surface modification approach is to redirect the microenvironment–host reaction toward regeneration.

Titanium is a popular implantable biomaterial due to its well-established biocompatibility and exceptional mechanical properties. Nevertheless, surface modification and functional coatings represent useful strategies for further enhancing its biological performance and introducing additional functionalities tailored to specific biomedical applications [3,4]. Changes in the surface properties of Ti implants tend to regulate the balance between pro- and anti-inflammatory cytokine expression by remodelling immune responses, favouring pro-healing regenerative processes [5,6]. Micro- and nano-structured Ti surfaces have been shown to modulate the early immune response by reducing M1 polarization and promoting the M2 macrophage phenotype, thereby supporting improved osseointegration [7]. However, this approach primarily relies on passive physico-chemical signals and lacks active biochemical signalling [8,9]. Recent studies in biomaterials research have highlighted the need for surface modification in composite coatings on Ti alloys to achieve multifunctionality and improve the properties of Ti substrates [10,11]. A considerable body of literature addresses immunomodulation and its impact on osteogenesis, in addition to the bone-forming effects of a variety of Ti implants [12,13,14,15,16].

Composite coatings based on biomimetic materials, such as nanohydroxyapatite (nHAp, Ca_10_(PO_4_)_6_(OH)_2_) and chitosan (Ch) derivatives, have demonstrated considerable potential in promoting mesenchymal stem cell (MSC) adhesion and proliferation while also modulating inflammatory responses [17,18]. Furthermore, bioactive coatings facilitate osteogenic differentiation, proliferation, and cell adhesion, while modestly shifting cytokine profiles toward anti-inflammatory responses [19,20]. Their immunomodulatory effect is largely indirect and primarily osteopromotive rather than strongly anti-inflammatory.

To further enhance biological functionality, incorporation of bioactive dopants has been explored as an effective strategy to actively regulate immune responses at the implant interface. Incorporation of functional ions, such as Zn, Sr, Cu, Mg or Se, enables active immunomodulation through controlled ionic release, promoting M2 macrophage polarization and coupling immune regulation with osteogenesis [6,16,20,21,22,23]. In this context, germanium (Ge) compounds have attracted increasing attention in biomaterials research due to their reported antioxidant, anti-inflammatory, and immunoregulatory properties [24]. In addition, the controlled release of Ge ions has been suggested to influence cellular signalling pathways involved in inflammation regulation and tissue regeneration, indicating its potential to improve the biological performance of implant surfaces [25,26]. Nevertheless, the incorporation of germanium into biomimetic, structurally controlled Ti coatings remains largely unexplored.

Notably, most immunomodulatory implant studies have primarily focused on macrophage-based models, giving insights into the complex cellular interactions occurring in the peri-implant microenvironment. A considerable body of literature has been published in this context, reflecting only one aspect of the complexity of the tissue–implant environment [27,28,29]. In contrast, MSCs have strong regenerative and paracrine capabilities and actively participate in early inflammatory signalling and immune cell interactions [30,31,32,33].

Through this research, we aim to contribute to solving the problem of the organism’s reaction to a foreign body, known as the foreign body response (FBR) [34]. One strategy for regulating the body’s response after implantation is to design implants based on anti-inflammatory materials [35]. Such implant-mediated immunomodulation strategies make it possible not only to influence FBR and inflammatory processes but also to promote more effective and higher-quality tissue regeneration [36].

The objective of this investigation is to design nanocomposite germanium (Ge)-enriched coatings based on nanohydroxyapatite (nHAp) particles and chitosan-oligolactate (ChOL, (C_12_H_24_N_2_O_9_)_n_) on Ti substrates (implants) with a potential for dual action—namely, with anti-inflammatory and osteoinductive properties. The anti-inflammatory and osteogenic potential of the developed coating was systematically evaluated using dental pulp stem cells (DPSCs) and compared with uncoated Ti surfaces to gain new insights into stem cell-mediated immune regulation at the bone–implant interface. We hypothesize that a synergistic combination of bioactive ceramic (nHAp), polymer (ChOL) and germanate (GeO_3_^2−^) components could create a favourable microenvironment at the implant–tissue interface, capable of simultaneously providing high biocompatibility, modulating the early inflammatory response, and promoting osteogenic differentiation.

## 2. Materials and Methods

### 2.1. Material Synthesis

A modified form of the previously optimized procedure [37,38,39,40,41] was used to prepare coatings on grade 2 commercially pure Ti containing amorphous calcium phosphate (ACP) (nHAp), ChOL, and germanate. The ACP powder was obtained by rapidly combining a 26.6 mass% Ca(NO_3_)_2_ × 4H_2_O solution (Sigma-Aldrich, St. Louis, MO, USA) with an (NH_4_)_3_PO_4_ solution prepared in double-distilled water. The latter solution was formulated from H_3_PO_4_ (85%, Sigma-Aldrich), NH_4_OH (28%, Sigma-Aldrich), and double-distilled water. After mixing and ageing, the resulting gel was collected, rinsed, and centrifuged for 1 h at 4000 rpm and 5 °C using a Hettich Universal 320 (Hettich GmbH, Tuttlingen, Germany). The obtained precipitate (ACP gel, Ca_x_H_y_(PO_4_)_z_·nH_2_O) was then freeze-dried under vacuum according to the procedure previously outlined in [6]. For subsequent in situ anaphoretic coating deposition, titanium plates (99.7% purity, ThermoFisher; 20 × 10 × 0.89 mm) were first sanded with SiC paper ranging from 600 to 3000 grit and then polished with alumina having grain sizes of 1, 0.3, and 0.05 μm (Buehler, Waukegan Road, Lake Bluff, IL, USA). The prepared plates were ultrasonically cleaned in 96% ethanol for 30 min at 120 W and 40 kHz using an ASONIC PRO 50 cleaner (ASonic, Ljubljana, Slovenia). To inhibit oxidation, the cleaned samples were kept in ethanol until use. The coatings were formed on the Ti substrates by in situ anaphoretic precipitation from an ethanolic suspension. To prepare this suspension, 277.8 mg of Na_2_GeO_3_, corresponding to 125 mg of Ge, was dissolved first. Chitosan oligosaccharide lactate (ChOL, Mw 5000, Sigma Aldrich, St. Louis, MO, USA) was then introduced in the same amount (125 mg), and the mixture was stirred overnight in accordance with established protocols. Once ChOL and Na_2_GeO_3_ had dissolved, 96% ethanol and ACP were incorporated, followed by another overnight stirring step. The pH was subsequently adjusted with 1 M NaOH. Anaphoretic deposition was carried out in a custom-built two-electrode electrochemical cell. A titanium plate served as the anode, whereas 316-grade stainless-steel plates (20 × 10 × 0.89 mm) were used as the cathode; the electrodes were positioned 10 mm apart. Deposition was performed at 60 V for 1 min, after which the coated samples were allowed to air-dry for 24 h at 25 °C. The resulting coating contained ACP (nHAp), ChOL, and germanate (GeO_3_^2−^) at a precisely adjusted mass ratio of 8:1:1 (nHAp:ChOL:Ge). During the anodizing/anaphoretic electrodeposition process, ACP is converted into the more stable crystalline HAp form, as demonstrated both in our earlier work and by other authors [37,42]. For presentation of the results, coated titanium is designated Ti/coating, whereas the uncoated titanium is designated Ti.

### 2.2. Physicochemical Characterization of Ti and Ti/Coating Sample

Surface morphology of the Ti and Ti/Coating samples was examined by field-emission scanning electron microscopy using a JEOL JSM-7610F Plus instrument (Tokyo, Japan). EDS spectra were processed with Aztec software (Version 6.3, Oxford Instruments, Oxfordshire, UK). FTIR measurements were obtained with a Michelson MB Series Bomen Fourier transform infrared spectroscope (Hartmann Braun, Munich, Germany), over the 400–4000 cm^−1^ wavenumber interval with a spectral resolution of 0.5 cm^−1^.

### 2.3. Drug Release Analysis

For release measurements, the disks were placed in 50 mL of complete growth medium at pH 7.4 and maintained at 37 °C for periods of up to 7 days. The total concentration of released Ge was determined by inductively coupled plasma optical emission spectrometry (ICP-OES). An iCAP 6500 Duo ICP instrument (Thermo Fisher Scientific, Cambridge, UK), operated with iTEVA software v5.8, was used for the measurements, and the samples entered the plasma by direct liquid aspiration. Calibration solutions spanning 1–50,000 μg/L were prepared from the certified Ge plasma standard solution SpecpureR (Alfa Aesar GmbH & Co, Karlsruhe, Germany).

### 2.4. In Vitro Cell Culture

Dental pulp stem cells (DPSCs) were isolated from a wisdom tooth and characterized as previously described [43]. Before the tooth was extracted, informed consent was obtained from a healthy patient, and the study was approved by the Ethics Committee of the University of Belgrade—School of Dental Medicine, Serbia. The DPSCs were maintained in complete growth medium (CM) composed of DMEM, 10% foetal bovine serum (FBS), and 1% antibiotic/antimitotic solution, with all components supplied by Thermo Fisher Scientific (Waltham, MA, USA). Cultures were kept at 37 °C in a humidified 5% CO_2_ atmosphere. CM was replaced every 2–3 days, and passaging was performed once cultures reached 80% confluence. Cells from passages 3–5 were used for the experiments.

### 2.5. Preparation of Treatment Media

Both Ti and Ti/coating disks were sterilized under UV light for 30 min on both sides. Individual disks were then placed for 24 h in 50 mL of either CM or, for osteogenic differentiation assays, osteogenic medium (StemMACS OsteoDiff Media, Miltenyi Biotec, Tokyo, Japan). This conditioning step was conducted at 37 °C in a humidified atmosphere containing 5% CO_2_, after which the disks were removed. The resulting treatment media were designated Ti and Ti/Coating, respectively. Cells maintained exclusively in CM were designated as the Control.

### 2.6. Microscopic Imaging

Scanning electron microscopy was used on both Ti and Ti/Coating surfaces to examine cell morphology, attachment, and distribution and to provide qualitative validation and context for the quantitative analyses that followed. Cells were seeded directly onto the disks at 5 × 10^4^ cells per disk and cultured for 24 h. After removal of the complete growth medium, the disks were gently rinsed with sterile phosphate-buffered saline (PBS). The samples were fixed in 4% paraformaldehyde for 20 min and then dehydrated sequentially in 30%, 50%, 70%, and 90% ethanol, with a 5 min incubation at each concentration. Before imaging, the samples were maintained in 100% ethanol, air-dried, and gold-sputtered for 2 min using a JFC 1100 ion sputter (Tokyo, Japan). SEM imaging was subsequently carried out on a JEOL JSM-7001F (JEOL Ltd., Tokyo, Japan) at an accelerating voltage of 5 kV.

### 2.7. MTT Assay

Cells were distributed into 96-well plates at 1 × 10^4^ cells per well. Each well then received 100 μL of the appropriate treatment medium, and incubation was continued for 24, 48, or 72 h. At the end of each treatment period, the supernatant was discarded and the wells were gently rinsed with PBS. A 100 μL volume of MTT solution (3-(4,5-dimethylthiazol-2-yl)-2,5-diphenyl tetrazolium bromide; Sigma-Aldrich, St. Louis, MO, USA) was added, followed by a 4 h incubation. The resulting formazan crystals were solubilized in 100 μL of dimethyl sulfoxide (DMSO; Sigma-Aldrich Inc., Burlington, MA, USA) under gentle agitation at 37 °C. Optical density (OD) was read at 570 nm on an RT-2100c microplate reader (Rayto, Guangming New District, Shenzhen, China). Cell viability (%) was calculated as (ODsample − ODblank)/(ODcontrol − ODblank) × 100.

### 2.8. LDH Assay

The CyQUANT™ LDH Cytotoxicity Assay was conducted in accordance with the manufacturer’s instructions. Cells were plated in 96-well plates at a density of 1 × 10^4^ cells per well and exposed to the different treatment media. For the maximum-release condition, cells were treated with lysis buffer to achieve complete cell lysis and, consequently, maximal LDH release. Cells maintained in complete growth medium (CM) constituted the control group.

### 2.9. Annexin V-FITC/PI Assay

For apoptosis analysis, cells were placed in 6-well plates at 1 × 10^5^ cells per well and cultured under the different treatment conditions. After 24 h, apoptosis was assessed with an Annexin V-FITC Apoptosis Detection Kit (Invitrogen, Thermo Fisher Scientific, Waltham, MA, USA) following the manufacturer’s instructions. Annexin V-FITC and propidium iodide (PI) staining was used to distinguish four cell populations: (i) viable cells (PI^−^/FITC^−^), (ii) early apoptotic cells (PI^−^/FITC^+^), (iii) late apoptotic cells (PI^+^/FITC^+^), and (iv) necrotic cells (PI^+^/FITC^−^). Data were acquired on a BD FACS Melody flow cytometer (BD Biosciences, San Jose, CA, USA) and processed with FlowJo software ver. 10.10.0 (Tree Star Inc., Ashland, OR, USA). At least 10,000 events were analyzed for each sample.

### 2.10. Flow Cytometric Analysis of Intracellular Reactive Oxygen Species (ROS)

DPSCs were plated in 6-well plates at 1 × 10^5^ cells per well and exposed to treatment for 24 h. The cells were then incubated for 30 min at 37 °C in the dark with 20 μM 2′,7′-dichlorofluorescin diacetate (DCF-DA; Sigma-Aldrich, St. Louis, MO, USA). A 100 μM H_2_O_2_ treatment was used as the positive control. Intracellular ROS was quantified from mean fluorescence intensity (MFI) measured with a BD FACS Melody flow cytometer, and the resulting data were evaluated using FlowJo software. A minimum of 10,000 events was acquired per sample.

### 2.11. Flow Cytometric Analysis of Immunomodulatory Markers

DPSCs were seeded into 6-well plates at 1 × 10^5^ cells per well and incubated for 24 h with the different treatment media. After treatment, the cells were collected and stained, in accordance with the manufacturer’s instructions, with fluorochrome-conjugated antibodies against CD120b (PE) and TNF-α (FITC) (MiltenyiBiotec, Bergisch Gladbach, Germany). Detection of intracellular TNF-α required fixation and permeabilization before staining, following the manufacturer’s protocol. Antibody staining was performed for 30 min at 4 °C in the dark. The cells were subsequently washed with PBS and resuspended for analysis. A BD FACS Melody flow cytometer was used for data acquisition, and FlowJo software was used for analysis. No fewer than 10,000 events were recorded for each sample. Marker expression was reported as mean fluorescence intensity (MFI), while representative histograms were used to show changes in fluorescence distribution among the experimental groups. TNF-α expression was assessed under standard culture conditions without secretion blockade, thereby reflecting physiologically relevant intracellular levels.

### 2.12. Osteogenic Differentiation and Alizarin Red Staining

One day after DPSCs had been seeded in 12-well plates at 5 × 10^4^ cells/well, each well received 1 mL of Ti or Ti/Coating treatment medium. Cultures were maintained for 7 days, with the medium replaced on day 3. Mineralized nodule formation was assessed by Alizarin Red S (ARS) staining. On day 7, cultures were fixed for 15 min in 4% neutral formalin and subsequently stained with 2% ARS (Sigma-Aldrich). For quantitative assessment of mineralization, the ARS-bound dye was released by incubating the cultures for 20 min with 250 μL of 1% hydrochloric acid in 70% ethanol. Absorbance of the extracted dye was recorded at 450 nm using an RT-2100c Microplate Reader (Rayto, Shenzhen, China). Cells grown in osteogenic medium served as the control.

### 2.13. RNA Extraction, cDNA Synthesis, and Quantitative PCR

RNA was isolated from cell samples containing 1 × 10^6^ cells per well in 6-well plates after 24 h of treatment, or after 7 days when osteogenic differentiation markers were analyzed. TRIzol reagent (Invitrogen, Thermo Fisher Scientific, Waltham, MA, USA) was used according to the manufacturer’s instructions. RNA concentration was measured with a microvolume spectrophotometer manufactured by Shimadzu Scientific Instruments (Carlsbad, CA, USA). For cDNA synthesis, 2 μg of total RNA was reverse-transcribed using an oligo d(T) primer and the Revert Aid First Strand cDNA Synthesis Kit (Thermo Fisher Scientific, Waltham, MA, USA) [44]. Real-time quantitative polymerase chain reaction (qPCR) mixtures contained 2 μL of first-strand cDNA, 0.75 μM of each forward and reverse primer, and 7.5 μL of SensiFAST SYBR Hi–ROX Kit (Bioline, London, UK). Gene expression was analyzed for the inflammatory markers TNFα, IL1β, IL6, COX2, and MAPK and for the osteogenic markers ALP, RUNX2, and BMP2. Glyceraldehyde-3-phosphate dehydrogenase (GAPDH) served as the reference gene. Gene-expression values were calculated with the 2^−∆Ct^ method [45]. The primer sequences used are listed in Table 1.

### 2.14. Statistical Analysis

Statistical analyses were carried out in GraphPad Prism version 9 (GraphPad Software, Inc., Boston, MA, USA). Distribution normality was examined with the Shapiro–Wilk test. Group differences were tested by ordinary one-way ANOVA, followed by Tukey’s post hoc multiple comparisons test. Results are reported as mean ± standard deviation (SD). Statistical significance is denoted as * *p* < 0.05, ** *p* < 0.01, *** *p* < 0.001, and **** *p* < 0.0001.

## 3. Results

### 3.1. Material Characterization

**Figure 1:**
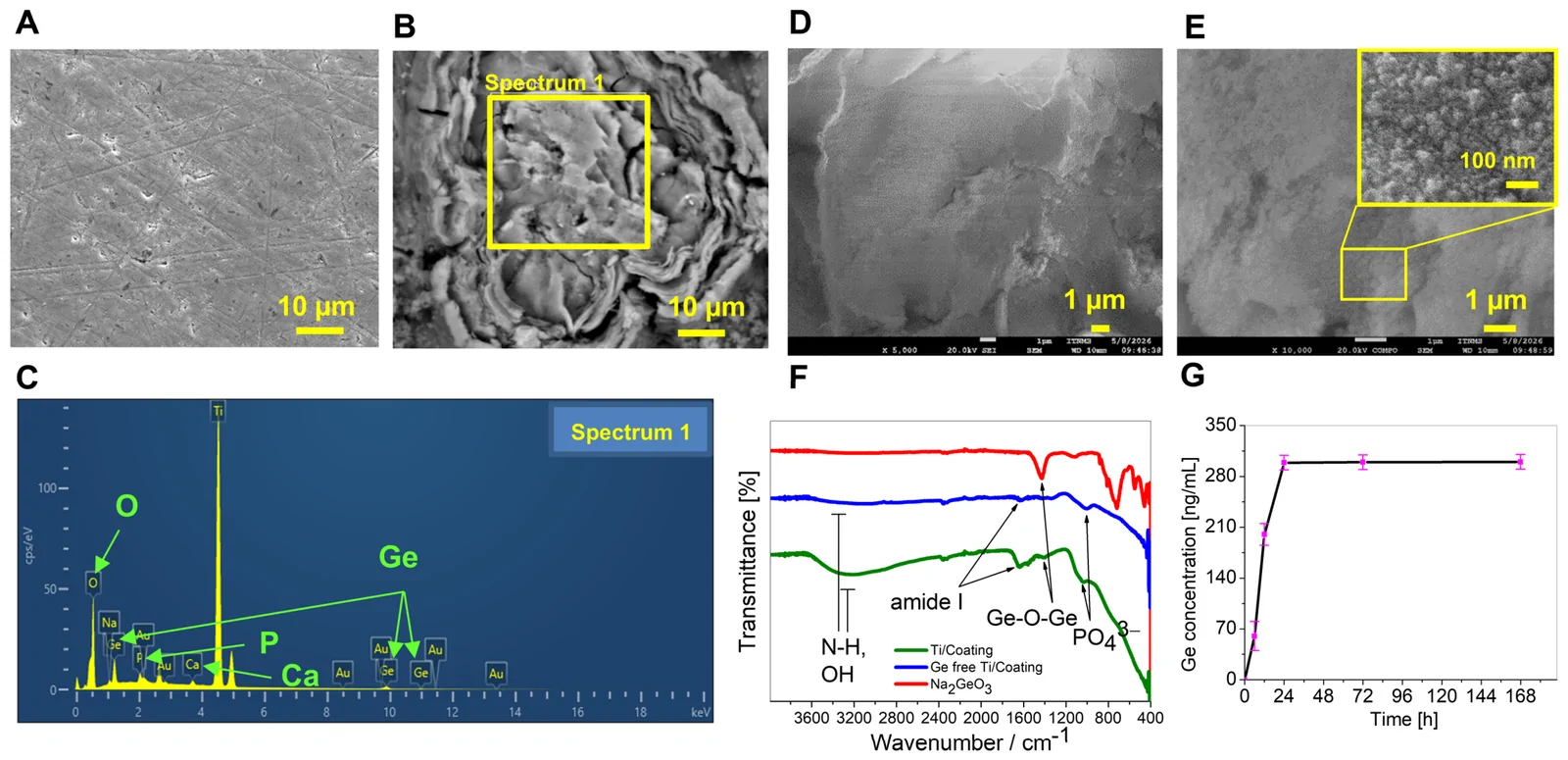
Morphology and base characterization of Ti and Ti/Coating samples: (**A**) SEM of Ti, (**B**) SEM of Ti/Coating, (**C**) the corresponding EDS spectra 1 of Ti/Coating, (**D**,**E**) SEM of Ti/Coating with magnifications 5000× and 10,000×, (**F**) FTIR spectra of Na_2_GeO_3_, Ge-free Ti/Coating and Ti/Coating and (**G**) over a seven-day period, the cumulative release curve of Ge from Ti/coating in medium at 37 °C.

Figure 1 depicts the morphology and basic characterization of Ti (Figure 1A) and Ti/Coating samples (Figure 1B). The EDS spectra of the marked Ti/Coating surface are shown in Figure 1C. Higher-magnification images of the Ti/Coating surfaces of 5000× and 10,000× are shown in Figure 1D,E. Successful coating of the Ti substrate was confirmed by FTIR analysis, as shown in Figure 1F. The release profile of Ge ions reveals an initial burst release within the first 24 h, reaching approximately 0.003 mg/mL, followed by a plateau with no significant increase over 7 days, indicating near-complete release (99 ± 1%) within this period (Figure 1G).

### 3.2. In Vitro Cellular Biocompatibility Assessment

#### 3.2.1. Morphological Assessment and Surface Cellular Adhesion

**Figure 2:**
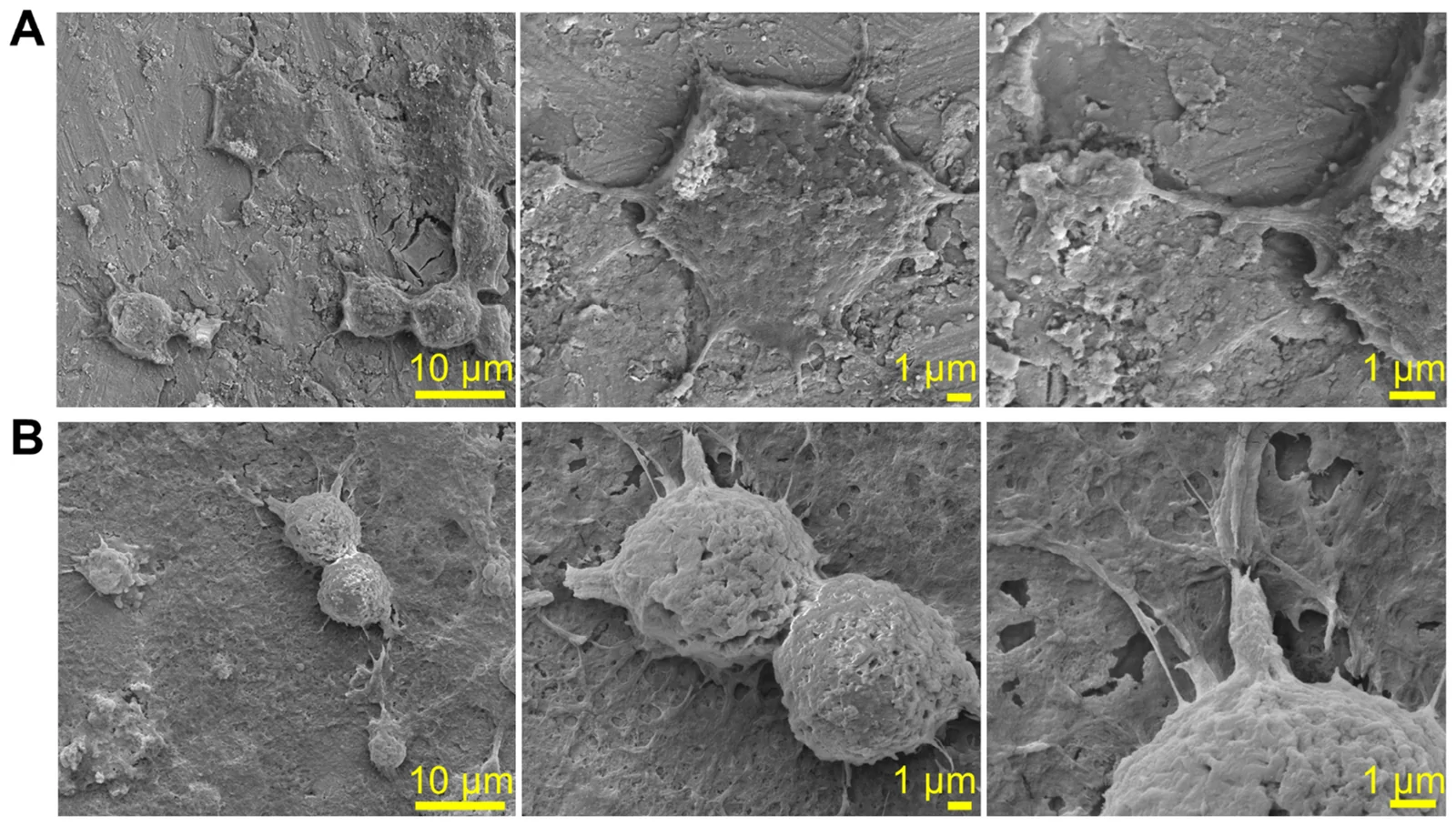
Representative SEM images of cells seeded onto (**A**) uncoated Ti and (**B**) Ti/Coating surfaces at 2000×, 5000× and 10,000× magnification. Scale bars: 10 µm (2000×) and 1 µm (5000× and 10,000×).

SEM analysis revealed normal cellular morphology and active cell proliferation on the material surfaces. As shown in Figure 2, DPSCs cultured on the Ti/Coating surface exhibited a well-spread morphology, with pronounced cytoplasmic extensions, including filopodia and lamellipodia, indicating strong cell attachment and preserved cellular integrity.

#### 3.2.2. Cell Viability of Ti and Ti/Coating and Cytotoxicity

**Figure 3:**
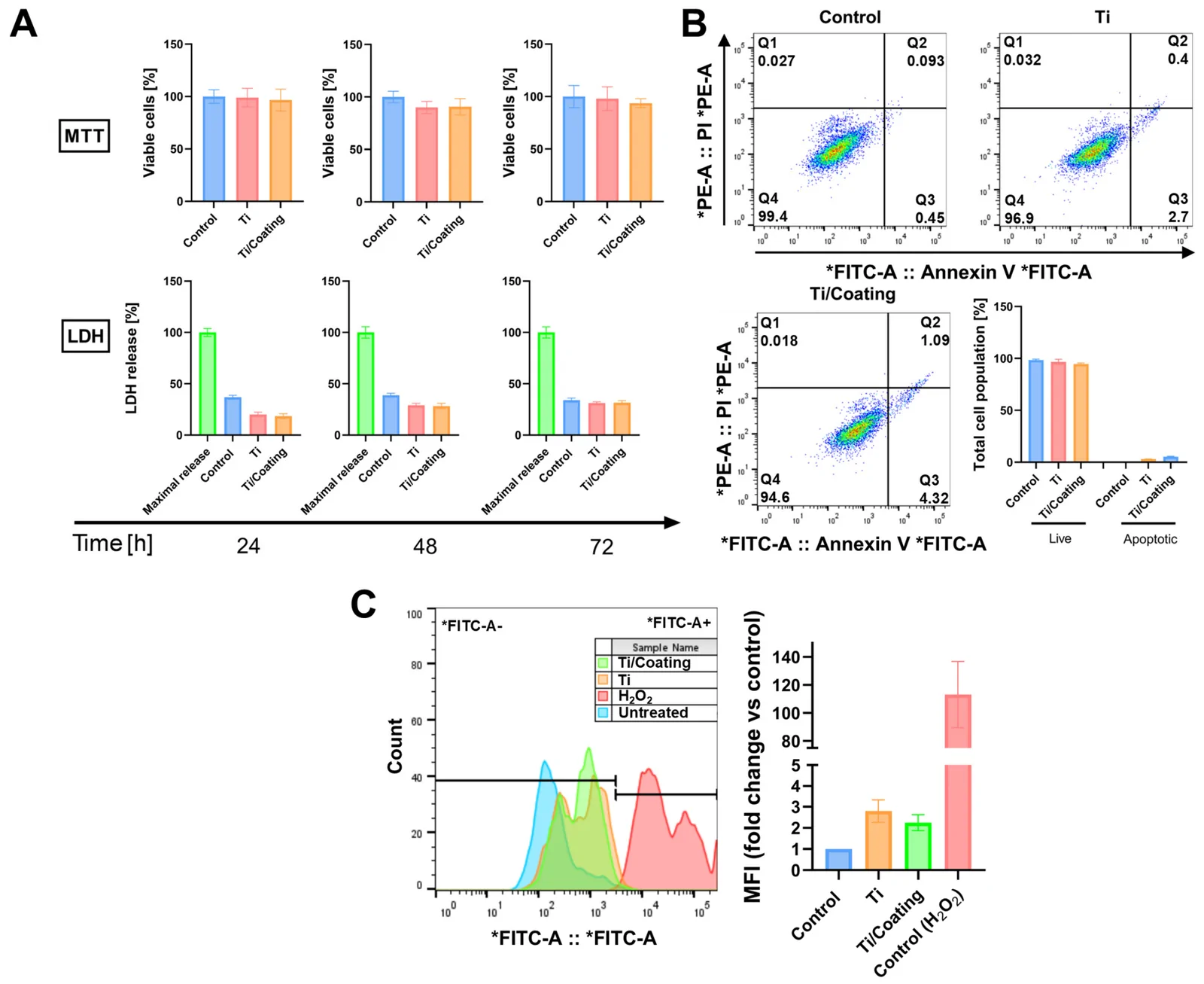
Effects of Ti and Ti/Coating on cell viability, apoptosis, and intracellular ROS levels. (**A**) MTT and LDH assays for biocompatibility evaluation. (**B**) Annexin V/PI apoptosis analysis with representative dot plots and quantification of live and apoptotic cell populations. (**C**) ROS analysis, with representative flow cytometry histograms and quantification of fluorescence intensity. Control—cells cultured in complete medium; Ti—cells treated with Ti-conditioned medium; Ti/Coating—cells treated with Ti/Coating-conditioned medium. Data are provided as the mean ± standard deviation (SD), *n* = 3.

No statistically significant differences in cell viability were observed among the experimental groups at any time point during the quantitative analysis of metabolic activity using the MTT assay (Figure 3A). After 24 h, mean viability values were 98.99 ± 8.82% for the Ti group and 96.65 ± 10.46% for the Ti/Coating group. At 48 h, viability remained high, with values of 90.00 ± 5.76% (Ti), and 90.65 ± 7.80% (Ti/Coating), respectively. Similarly, after 72 h, mean viability was 98.01 ± 11.31% for Ti and 93.70 ± 4.22% for Ti/Coating. Although slight reductions in metabolic activity were observed in treated groups at certain time points, all values remained 90% and above, indicating preserved mitochondrial activity and confirming good biocompatibility of the tested materials.

Additionally, LDH release was evaluated to assess membrane integrity and cytotoxicity at 24, 48, and 72 h (Figure 3A). The untreated control group showed LDH levels of 36.88 ± 1.99%, 38.84 ± 1.99%, and 34.03 ± 2.00% at 24, 48, and 72 h, respectively. In comparison, the Ti group exhibited lower LDH release at all time points (20.12 ± 2.15%, 29.05 ± 1.86%, and 31.36 ± 1.02%), as did the Ti/Coating group (18.52 ± 2.32%, 28.34 ± 2.55%, and 31.58 ± 1.93% at 24, 48, and 72 h, respectively). Notably, LDH levels in both Ti and Ti/Coating groups were consistently lower than or comparable to those of the control group, indicating preserved membrane integrity and the absence of cytotoxic effects.

#### 3.2.3. Apoptosis Assessment by Annexin V/PI Staining

Annexin V/PI staining demonstrated a high proportion of viable cells across all experimental groups after a 24-h treatment (Figure 3B). The percentage of live cells was 98.42 ± 0.85% in the control group, 96.60 ± 2.56% in the Ti group, and 94.60 ± 1.00% in the Ti/Coating group. Although a slight decrease in the percentage of viable cells was observed in the treated groups, all values remained above 90%, indicating preserved cell viability. Consistently, the proportion of apoptotic cells (early + late apoptosis) remained low, with values of 3.10 ± 0.10% for the Ti group and 5.41 ± 0.41% for Ti/Coating group. Despite a mild increase in apoptosis in the treated groups, these levels remained minimal and were not indicative of significant cytotoxic effects.

#### 3.2.4. Intracellular ROS Production Analysis

Intracellular ROS levels were quantified as fold changes in mean fluorescence intensity (MFI) relative to the control group (cells cultured in complete growth medium). Compared with the control group, the Ti and Ti/Coating groups exhibited a moderate increase in ROS levels (~3.09-fold and ~2.44-fold, respectively) (Figure 3C). Notably, ROS generation in the Ti/Coating group was lower than that observed in the Ti group, suggesting a potential modulatory effect of the coating on oxidative stress. Despite this increase, ROS levels in both material groups remained substantially lower than those induced by 100 µM H_2_O_2_, indicating the absence of excessive oxidative stress.

### 3.3. Immunomodulatory Effects of Ti and Ti/Coating on DPSCs

#### 3.3.1. Gene Expression Analysis of Pro-Inflammatory Markers

**Figure 4:**
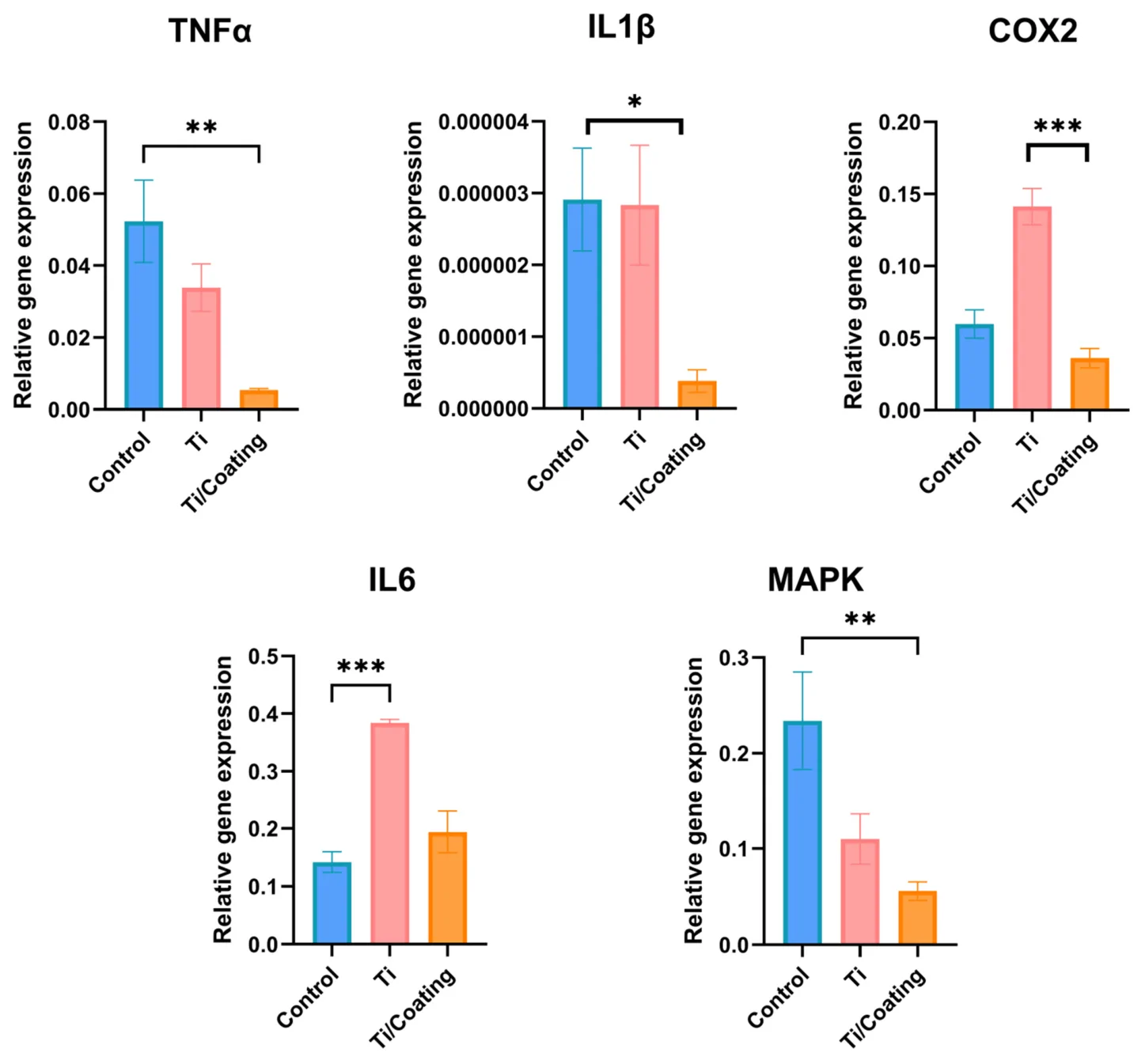
Effects of Ti and Ti/Coating on inflammation-related gene expression (TNF-α, IL-1β, COX-2, IL-6, and MAPK) after 24 h of treatment. Control—cells cultured in complete growth medium; Ti—cells treated with Ti-conditioned medium; Ti/Coating—cells treated with Ti/Coating-conditioned medium. Data are provided as the mean ± standard deviation (SD), *n* = 3. * *p* < 0.05, ** *p* < 0.01, and *** *p* < 0.001 indicate statistical significance.

The relative expression of inflammatory genes was analyzed by real-time PCR. The results demonstrated a downregulation of the pro-inflammatory genes (TNF-α, IL-1β, COX-2, IL-6, and MAPK) in cells treated with Ti/Coating compared with both the control group and cells exposed to Ti, suggesting a shift toward an anti-inflammatory cellular phenotype (Figure 4). qPCR analysis revealed downregulation of key pro-inflammatory genes following Ti/Coating treatment, including TNF-α (9.45-fold vs. control; 6.25-fold vs. Ti), IL-1β (7.69-fold vs. control; 7.46-fold vs. Ti), COX-2 (1.66-fold vs. control; 3.92-fold vs. Ti), IL-6 (1.98-fold vs. Ti), and MAPK (4.18-fold vs. control; 1.96-fold vs. Ti).

#### 3.3.2. Flow Cytometry Analysis of Pro-Inflammatory Markers Following Ti/Coating Treatment

**Figure 5:**
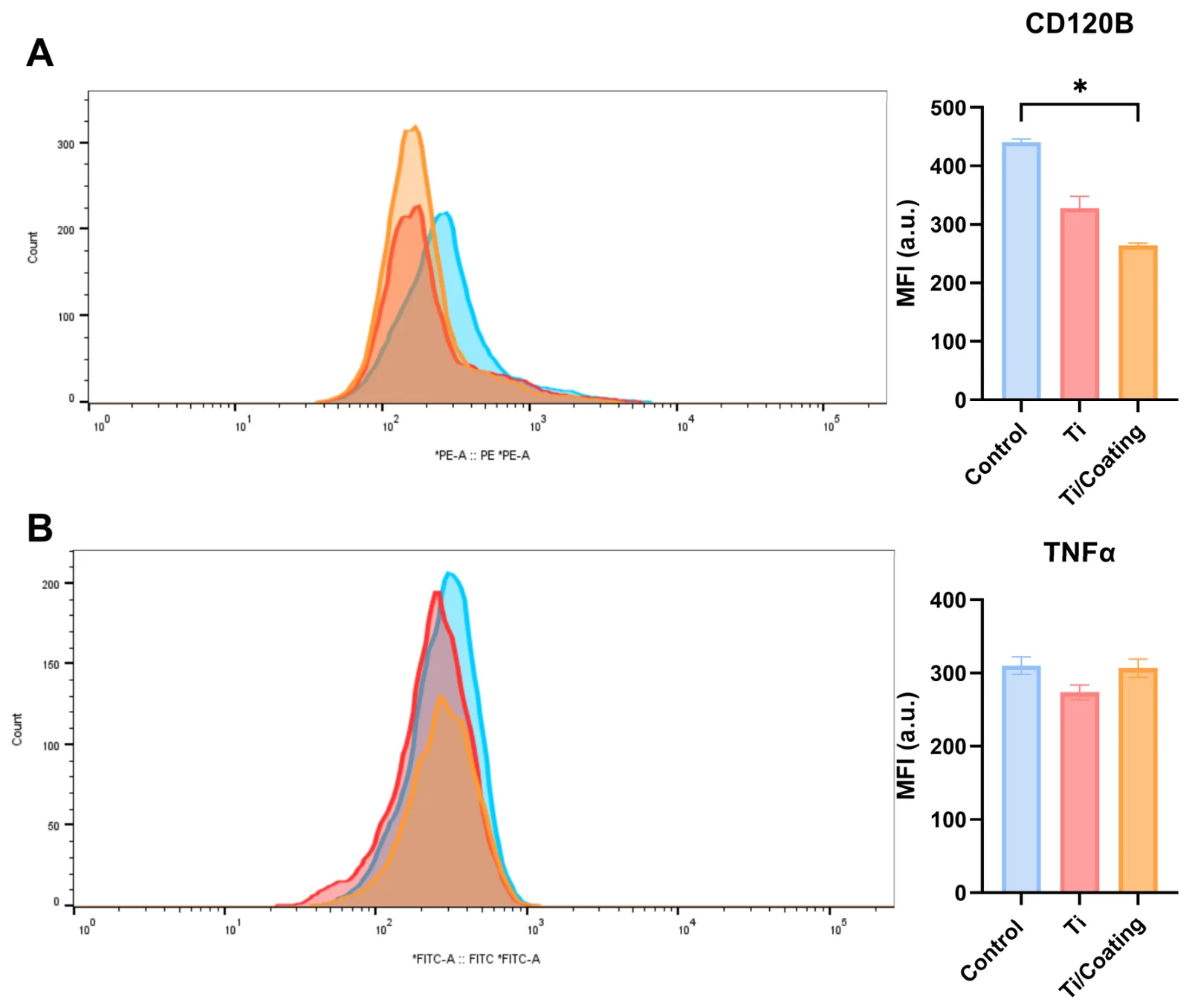
Flow cytometry analysis of surface (**A**) CD120B and (**B**) intracellular TNF-α markers. Colors on the representative histograms: blue—control; red—Ti group; orange—Ti/Coating group. Data are provided as the mean fluorescence intensity (MFI) ± standard deviation (SD), *n* = 3. * *p* < 0.05 indicates statistical significance.

Flow cytometry analysis was performed to evaluate the immunomodulatory response of cells cultured with Ti and Ti/Coating compared to the control group. Marker expression was quantified as mean fluorescence intensity (MFI), reflecting relative expression levels per cell. The expression of pro-inflammatory surface marker CD120b (TNFR2) (Figure 5A) was significantly reduced in Ti/Coating group compared with the control group (*p* < 0.05). Intracellular TNF-α expression (Figure 5A) did not show a substantial reduction in the Ti/Coating group compared to Ti, remaining comparable or slightly elevated relative to the control group. Histogram profiles of both markers showed largely overlapping populations without distinct positive subpopulations.

### 3.4. Evaluation of DPSC Osteogenic Differentiation Following Ti and Ti/Coating Treatment

#### 3.4.1. Assessment of Matrix Mineralization by Alizarin Red S Staining

**Figure 6:**
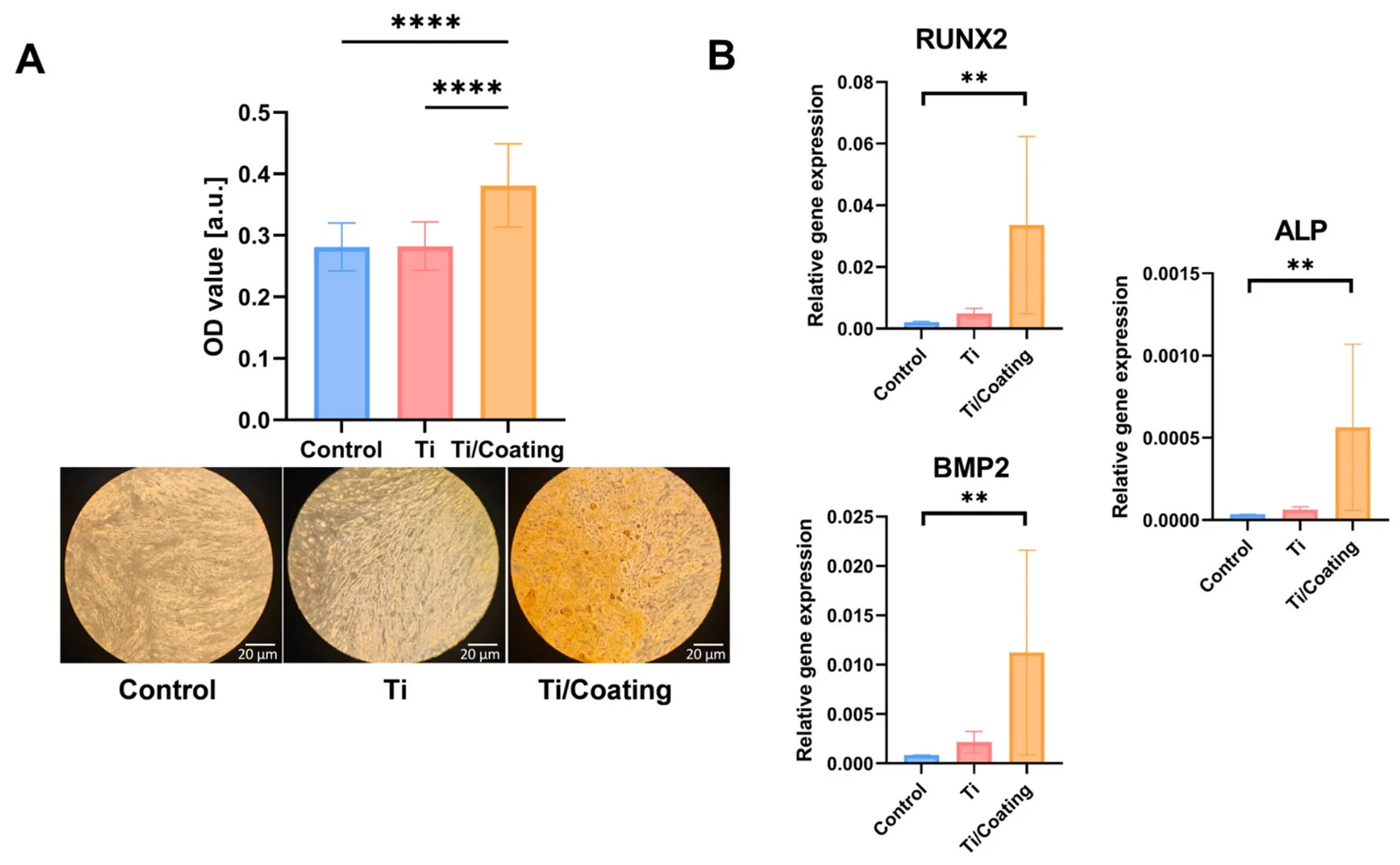
Effects of Ti and Ti/Coating on osteo-differentiation: (**A**) Alizarin Red S staining and quantification; (**B**) early osteogenic marker expression analysis. Control—cells cultured in osteogenic medium; Ti—cells treated with Ti-conditioned medium; Ti/Coating—cells treated with Ti/Coating-conditioned medium. Data are provided as the mean ± standard deviation (SD), *n* = 3. ** *p* < 0.01, and **** *p* < 0.0001 indicate statistically significant differences.

Alizarin Red S staining was implemented to assess mineralized matrix formation as an indicator of osteogenic differentiation (Figure 6A). Qualitative analysis revealed a markedly increased intensity of ARS staining in the Ti/Coating group than in both Ti and control groups, indicating enhanced calcium deposition and mineralized nodule formation. In contrast, the control and Ti groups exhibited minimal staining.

#### 3.4.2. Gene Expression Analysis of Osteogenic Markers

The expression of genes associated with osteogenesis was assessed using quantitative real-time PCR to further assess the osteogenic potential of the Ti/Coating system (Figure 6B). The results demonstrated an upregulation of key osteogenic markers, including alkaline phosphatase (ALP), runt-related transcription factor 2 (RUNX2), and bone morphogenetic protein 2 gene (BMP2), in the Ti/Coating group compared to both Ti and control groups. Among these, RUNX2, as an early transcriptional regulator of osteogenic differentiation, showed increased expression, indicating activation of osteogenic commitment. This was accompanied by elevated ALP and BMP2 expression, reflecting enhanced early-stage osteoblastic activity.

## 4. Discussion

### 4.1. Substrate (Ti) Surface Modification/Coating

It is notable that the Ti surface (Figure 1A) is significantly smoother than the Ti/coating (Figure 1B) surface, as expected. The O, P and Ca originate from calcium phosphate particles, specifically HAp (Figure 1C). As already mentioned (in Section 2.1), during electrodeposition, the ACP phase transformed into a more stable form of calcium phosphate, specifically HAp (with particle sizes smaller than 100 nm), as established in our previous research [37,39,40]. The FTIR spectra (Figure 1F) of the investigated coatings provide a detailed insight into the structure of the matrix and reveal additional spectral features that confirm the incorporation of germanate (GeO_3_^2−^). Beyond the main absorption bands, particular attention must be given to the appearance of shoulders—subtle features not present in the spectrum of the Ge-free coating—which is an important indicator of structural modification. The Ge-free coating shows the bands typical of chitosan–calcium phosphate systems. Bands at approximately 1650 cm^−1^ (amide I) and 1590–1550 cm^−1^ (amide II) confirm the presence of chitosan, while the broad band at 3200–3500 cm^−1^ corresponds to O–H and N–H stretching vibrations [46,47]. The phosphate phase is identified by a broad ν_3_(PO_4_^3−^) band at ~1000–1100 cm^−1^ and bending modes at ~560 and ~600 cm^−1^. In contrast, the Ti/coating retains all major matrix bands but exhibits additional shoulders and band asymmetries, particularly in regions associated with Ge vibrations. These shoulders are not observed in the Ge-free Ti/Coatings spectrum and therefore provide key evidence of GeO_3_^2−^ incorporation. A distinct shoulder at ~1430 cm^−1^ appears in the Ti/Coating sample. This feature is absent in the Ge-free Ti/Coating sample and corresponds to asymmetric stretching vibration modes of Ge–O–Ge bonds, which are well documented in germanate systems [48,49].

### 4.2. Ti and Ti/Coating’s Impact on Cellular Viability

In our study, the biocompatibility of the Ti and Ti/Coating system was comprehensively evaluated through morphological and functional assays. Scanning electron microscopy analysis (Figure 2) demonstrated that DPSCs cultured directly on the Ti/Coating surface maintained a well-spread morphology, marked by an organized cytoskeletal architecture and preserved cell to cell junction and adhesion. These morphological features are indicative of normal cell behaviour and are essential for maintaining cellular integrity for further regenerative applications [50]. In addition, the cells exhibited signs of active division, indicating that the Ti/Coating supports cell coherence and structural integrity.

Functional assays provided insight into cytocompatibility. The MTT assay demonstrated that the metabolic activity of DPSCs remained unchanged following exposure to Ti/Coating, with cell viability consistently exceeding the 70% threshold defined by the ISO 10993-5 standard [51], thereby confirming preserved mitochondrial function and the non-cytotoxic nature of the material (Figure 3A). In agreement with these findings, LDH release remained below 32%, indicating preserved plasma membrane integrity and the absence of significant cytotoxic effects, thus fulfilling baseline cytocompatibility criteria for biomaterials (Figure 3A). Similar results have been reported for comparable biopolymer coatings in human osteoblasts (hFOB 1.19) [52].

Consistent with these results, the Annexin V analysis revealed a predominance of live cells population, indicating that apoptosis was not activated in the DPSC population following Ti/Coating treatment for 24 h (Figure 3B). The lack of apoptosis is particularly important for stem cells, as apoptotic signalling can compromise regenerative capacity even in the absence of overt cytotoxicity [53].

Furthermore, intracellular ROS levels remained within a moderate range, indicating that the Ti/Coating system does not induce cellular stress or compromise redox balance (Figure 3C). ROS generation in the Ti/Coating group was lower than that observed in the Ti group, which is consistent with previously reported antioxidant properties of germanium-based materials [25].

### 4.3. The Influence of Ti and Ti/Coating on Pro-Inflammatory Markers

The inflammatory response of DPSCs following Ti and Ti/Coating treatment was assessed by analyzing the expression of key pro-inflammatory genes (Figure 4). Tumor necrosis factor alpha (TNF-α) and interleukin 1 beta (IL-1β) are early-response core pro-inflammatory cytokines that initiate and amplify inflammatory signalling, while interleukin 6 (IL-6) acts as a key mediator linking acute inflammation with tissue remodelling processes such as simulation of bone resorption [54,55]. Cyclooxygenase-2 (COX2) is an inducible enzyme responsible for prostaglandin synthesis and is commonly associated with inflammatory progression [56]. In parallel, mitogen-activated protein kinase (MAPK) signalling acts as a central regulatory pathway controlling the transcription of multiple pro-inflammatory genes in response to extracellular stimuli [57,58]. Given that pro-inflammatory cytokines are often associated with cytotoxicity, oxidative stress, and impaired regeneration [59], the suppressed inflammatory gene profile further supports the immunocompatibility and therapeutic potential of the developed Ti/Coating system. Taken together, it is most likely that the Ti/Coating system has a combined inhibitory effect on key inflammatory signalling cascades regulating genes, particularly those governed by MAPK, resulting in the coordinated downregulation of TNF-α, IL-1β, IL-6, and COX2 observed in this study. Most likely, this effect is due to the synergistic contribution of the Ti/Coating components. HAp has been associated with reduced secretion of pro-inflammatory cytokines secretion, such as TNF-α, IL-1β and IL-6, while promoting regenerative factors [60,61]. Ch can attenuate inflammatory responses by binding pro-inflammatory mediators and reducing downstream signalling, although under certain conditions it may also activate inflammatory pathways [62]. Ge and its compounds have been reported to reduce the expression of pro-inflammatory cytokines, including TNF-α, IL-1β, and IL-6, through attenuation of oxidative stress and regulation of specific signalling mechanisms contributing to a reduction in the inflammatory cell infiltration [25].

**Figure 7:**
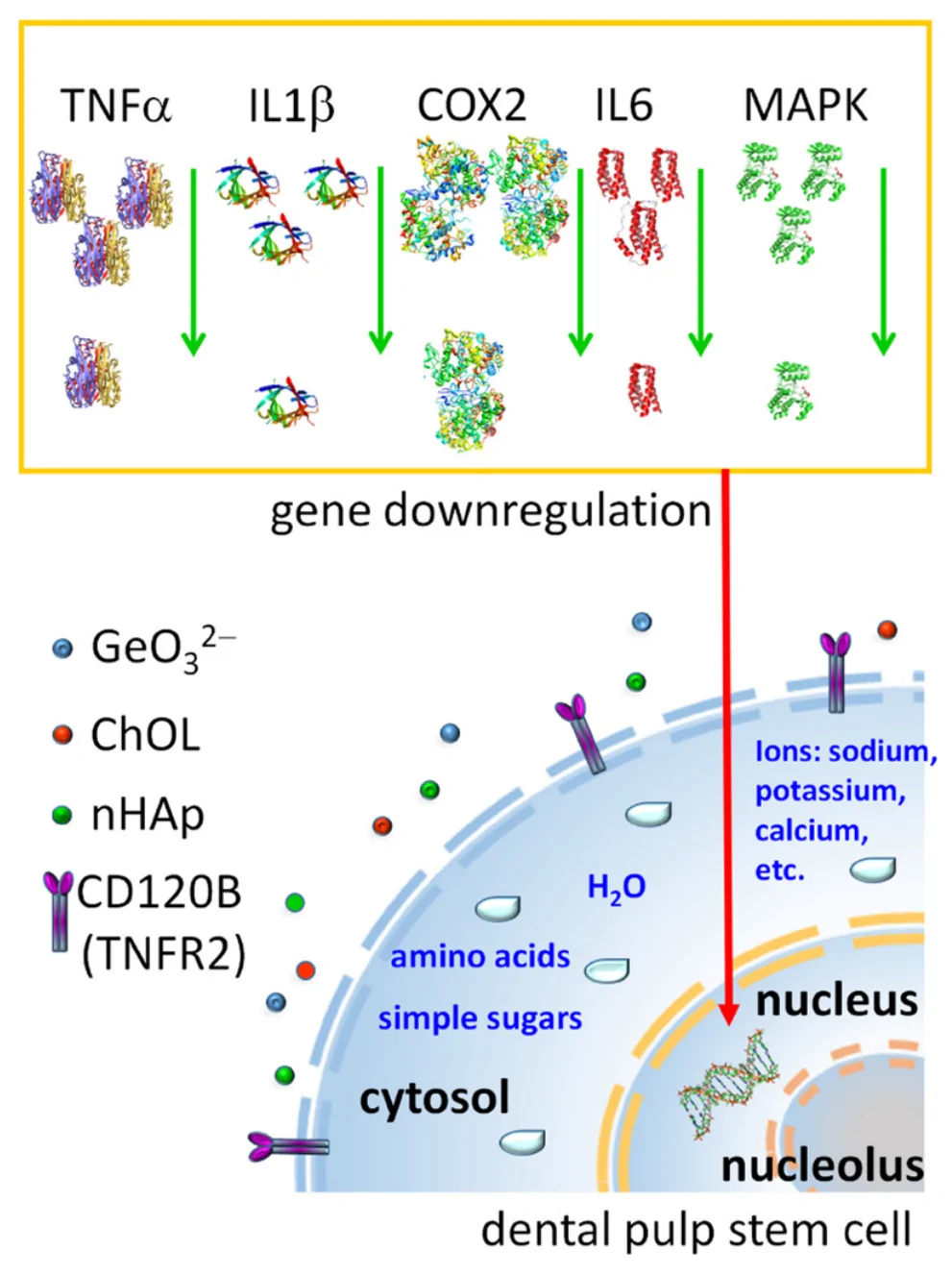
Schematic diagram of the proposed immunomodulatory mechanism of Ti/Coating in DPSCs.

The qPCR findings are further supported by flow cytometry analysis of surface and intracellular immunomodulatory markers (Figure 5). The downregulation of CD120B (TNFR2) suggests an alteration in TNF-related signalling at the receptor level rather than a direct suppression of cytokine production. In regenerative contexts, TNFR2 has been associated with pro-survival and immunomodulatory functions [63]. Therefore, the observed reduction in TNFR2 expression on the Ti and Ti/Coating may reflect an altered TNF signalling balance rather than simple anti-inflammatory suppression (Figure 7). Given that intracellular TNF-α levels were not markedly reduced, Ti/Coating appears to influence receptor-level responsiveness more than cytokine production [64]. In this context, the modest reduction in activation markers without complete cytokine inhibition may indicate a fine adjusting effect, rather than evident immunosuppression. This selective modulation could potentially support regenerative processes while avoiding excessive inflammatory damage [65]. Overall, these results indicate that the Ti/Coating system induces a selective immunomodulatory effect, marked by attenuation of excessive activation while preserving essential inflammatory signalling. This controlled modulation could be considered beneficial for maintaining a microenvironment that supports tissue regeneration and implant integration.

### 4.4. The Influence of Ti/Coating on Osteogenic Differentiation

In DPSCs, reduced inflammatory gene and protein expression is closely associated with enhanced regenerative capacity, supporting the potential of the Ti/Coating system for tissue engineering applications, particularly in promoting osteogenic differentiation [66,67,68,69]. The osteoinductive potential of DPSCs was evaluated through osteogenesis-related markers: *ALP*, *RUNX2*, and *BMP2*. *ALP* is an early osteogenic marker with a critical role in bone matrix mineralization, primarily through increasing local inorganic phosphate availability during osteoblast maturation [70]. RUNX2 and *BMP2* are early-stage markers governing osteoblast differentiation by regulating the expression of multiple osteogenesis-related genes [71]. Among the coating components, hydroxyapatite (HAp), as a bioactive and osteoconductive material, is well known to promote osteogenesis [72]. Previous studies have demonstrated that nHAp particles can enhance osteogenic differentiation, partly by acting as carriers for bone morphogenetic protein (BMP)-mediated signalling [73,74]. Similarly, chitosan, a biocompatible polymer, supports osteogenesis by facilitating mineralized matrix formation and increasing ALP activity [75]. Furthermore, germanium-containing compounds have been reported to enhance osteoinductive responses [76]. Taken together, the synergistic effect of these components within the Ti/Coating system likely contributes to the observed upregulation of all four osteogenesis-related genes, indicating a strong osteoinductive effect and supporting its potential application in bone and dental tissue regeneration.

This study has several limitations. Initially, the investigations were conducted under in vitro conditions, which do not entirely replicate the complexity of the in vivo implant microenvironment. Only DPSCs were used, whereas implant integration involves interactions with multiple cell types, including immune and endothelial cells. Although immunomodulatory effects were evaluated through selected gene and protein markers, a detailed analysis of the underlying signalling pathways was not performed. Additionally, discrepancies between gene and protein expression levels (TNF-α) may reflect differences in transcriptional and post-transcriptional regulation, which were not further investigated. Furthermore, the study focused on short-term responses, while long-term biological effects, as well as in vivo validation, remain to be addressed. An additional limitation is the absence of a Ge-free nHAp/ChOL coating as a separate biological comparison group. The present study was designed to evaluate the biological performance of the complete multicomponent coating relative to uncoated Ti and, therefore, does not allow the individual contribution of Ge to be distinguished from the combined effects of the nHAp/ChOL matrix. Direct comparison of otherwise identical Ge-free and Ge-containing coatings should be addressed in future studies aimed specifically at resolving the contribution of individual coating components.

## 5. Conclusions

A multifunctional germanium-enriched Ti/Coating was successfully developed, demonstrating excellent cytocompatibility toward DPSCs. The coating preserved cell viability and function while inducing a selective immunomodulatory effect, characterized by downregulation of pro-inflammatory gene expression and reduced TNFR2 levels. In addition, Ti/Coating significantly enhanced early osteogenic differentiation of DPSCs, as evidenced by increased mineralization and upregulation of osteogenic markers. These findings indicate that the developed coating promotes a favorable regenerative microenvironment, highlighting its potential for improving implant integration. Additional in vivo studies are necessary to verify its clinical applicability.

## Figures and Tables

**Table 1:** Primers with corresponding sequences used in the study.

Product Name		Sequences (5′→3′)
*RUNX2*	Forward Reverse	ACAAACAACCACAGAACCACAAGT GTCTCGGTGGCTGGTAGTGA
*ALP*	Forward Reverse	CCACGTCTTCACATTTGGTG ATGGCAGTGAAGGGCTTCTT
*BMP2*	Forward Reverse	CACTGTGCGCAGCTTCC CCTCCGTGGGGATAGAACTT
*TNFα*	Forward Reverse	CTCTTCTGCCTGCTGCACTTTG ATGGGCTACAGGCTTGTCACTC
*IL1β*	Forward Reverse	CCACAGACCTTCCAGGAGAATG GTGCAGTTCAGTGATCGTACAGG
*IL6*	Forward Reverse	AGACAGCCACTCACCTCTTCAG TTCTGCCAGTGCCTCTTTGCTG
*COX2*	Forward Reverse	CGGTGAAACTCTGGCTAGACAG GCAAACCGTAGATGCTCAGGGA
*MAPK*	Forward Reverse	ACACCAACCTCTCGTACATCGG TGGCAGTAGGTCTGGTGCTCAA
*GAPDH*	Forward Reverse	TCATGACCACAGTCCATGCCATCA CCCTGTTGCTGTAGCCAAATTCGT

## Data Availability

The original contributions presented in this study are included in the article. Further inquiries can be directed to the corresponding authors.

## Abbreviations

The following abbreviations are used in this manuscript:

DPSC	dental pulp stem cell
MTT	3-(4,5-dimethylthiazol-2-yl)-2,5-diphenyltetrazolium bromide
LDH	lactate dehydrogenase
nHAp	nanohydroxyapatite
ChOL	chitosan-oligolactate
GeO_3_^2−^	germanate
FBR	foreign body response
MSC	mesenchymal stem cell
ACP	amorphous calcium phosphate
FTIR	Fourier transform infrared spectroscope
ICP-OES	inductively coupled plasma optical emission spectrometry
CM	complete growth medium
FBS	foetal bovine serum
PBS	phosphate-buffered saline
DMSO	dimethyl sulfoxide
ARS	Alizarin Red S
ROS	reactive oxygen species
